# Developing a virtual patient: design, usability, and learning effect in communication skills training

**DOI:** 10.1186/s12909-023-04860-7

**Published:** 2023-11-22

**Authors:** Baris Sezer, Tufan Asli Sezer, Gulsen Tasdelen Teker, Melih Elcin

**Affiliations:** 1 Faculty of Medicine, Department of Medical Education and Informatics, Hacettepe University, Altindag, Ankara, Turkey; 2 Faculty of Nursing, Department of Pediatric Nursing, Ankara University, Ankara, Turkey

**Keywords:** History-taking, Technology-enhanced teaching, Simulation, Communication, Virtual human

## Abstract

**Background:**

Literature shows that Virtual Patients (VPs) find extensive usage in the field of health sciences, especially in the post-pandemic period. VPs are successfully utilized in developing various effective skills like medical interview. However, this technology is quite new in Turkey and has not yet been used in communication skills training in a structured form. This research aimed to develop a virtual patient to improve the communication skills of medical students.

**Methods:**

Developmental research method was used in the study. The implementation phase involved the one group posttest quasi-experimental design. The study group comprised of experts in various fields and 213 medical students. Needs Analysis Form, Scenario Building Form, System Validation Form, Communication Skills Assessment Form, and Interview Form were used as data collection tools. The research primarily concentrated on ensuring minimal errors within the system and enhancing students’ communication skill scores.

**Results:**

The study found that VP was effective in teaching communication skills. Communication skills improved from a mean score of 36.74 in the first interview with 15 students to 74.2 in the final application with 198 students. It was determined that the students who practiced repeatedly (n = 26) made 17% more effective interviews than their first practices (score: 89.2). The script matching of the VP was 83%. Other data obtained from the students generally showed that the VP application was developed in accordance with the purpose, that it was user-friendly, and that the scenarios were adequate.

**Conclusion:**

VPs like this have the potential to develop skills such as history taking, clinical reasoning, etc., which are very important in the field of health sciences.

## Background

Medical knowledge, problem-solving, physical examination, and communication skills are the four key components of competencies that make up good clinical practice [1]. Communication skills form the fundamental basis of clinical practice, and without acquisition of this skill, the other components significantly lose their meaning [2]. The history-taking skill, which is the core of communication skills, enables making clinical decisions by receiving accurate information about the patient, usually directly from the patient, and in some cases from the patient’s relatives and other health personnel [3].

Communication skills training in medical education programs is given limitedly, in structured and applied form, in Turkey. Various methods and approaches such as lectures, bedside demonstration, script-based teaching, role-playing, use of standardized patients (SPs), and clinical practice are generally utilized in these trainings [3]. Bedside demonstrations and classroom lectures are methods that proceed in the form of didactic narration in which students are not active. There is no verbal or non-verbal interview skill teaching in case scripts used to develop these skills of the students structurally. Although clinical practice is one of the best ways, it does not apply to preclinical students who do not have the chance to see enough patients. Role-playing (generally peer) is not as effective as SPs due to the lack of experience of students [3]. Since the 1960s, SPs in the biological simulation category have been used more frequently and effectively in training these skills [4]. The standardized patient is “a healthy individual trained to accurately and consistently describe a specific history of illness and to evaluate student performance” [5]. This SP use has many positive contributions such as ‘creating real-life learning environment’, ‘supplying synchronous interactivity’, and ‘giving instant feedback’ as well as some limitations such as ‘objectivity’, ‘consistency’, ‘standardization’ etc. [6, 7]. Especially in situations where there are crowded student communities, these limitations (forgetting the script, cost, fatigue, bias, etc.) are much more prominent when SPs interview a large number of students [8].

To achieve the ideal evaluation of the teaching history-taking ability, the teaching environment should reflect the real situation as much as possible and the differences in practice between peers should be minimized [9]. It is important to use standardized and objective evaluation methods for students’ history-taking skills. When the student performance is evaluated in the communication skills training carried out with SPs, in some cases, the SPs give feedback to the students after the interview. This situation may reveal a bias due to the human variability and error problem [5]. In some cases, the interviews of the students are recorded in video and the trainers evaluate the students in the following sessions. However, in situations with a large number of students, it is difficult timewise to watch each student’s record and provide feedback. Virtual patients (VPs) have the potential to be a solution to this problem [8, 10, 11].

The VP, which falls under the category of virtual human, is a computer application that simulates real-life clinical scenarios in which the learner acts as a healthcare provider, and is used for training on therapeutic decision-making, diagnosis, physical examination, and history-taking [12, 13]. VPs are being successfully used in developing various effective skills like medical interview [8, 11, 14], developing empathy [15, 16], the expression of medical errors to other medical personnel [17, 18], diagnostic questioning skills [19], and leadership skills [20]. The most important advantages are ‘objectivity’, ‘standardization’, ‘ability to choose agents suitable for the scenario’, ‘logging’, and ‘repetitive practicing’ [21]. A VP application can keep records of students’ ability to take histories without a number limit; and by evaluating them accordingly, it can present the degree of achievement of learning objectives to the student/trainer based on evidence.

Literature shows that the VP application is widely used in health sciences, especially in the post-pandemic period. However, this concept is quite new in Turkey and has not yet been used in communication skills training in a structured form. In the literature, it is reported that VP applications developed for communication skills should be supported with appropriate scenarios, should offer formative evaluation/scoring of skills, should make rich speech detection, and give detailed feedback at the end of use [9, 21]. In this regard, this research (supported by TUBITAK – No: 318S220) aims to design, create, and assess an efficient VP application enriched with various scenarios (headache, kidney pain, ear pain, stomach pain, digestive problem, and cold) for medical students.

## Method

This study was planned and carried out as developmental research. Developmental research is defined as “the systematic design, development, and evaluation of instructional programs, processes or products” [22]. The generic development cycle with “Analysis, Design, Development, Implementation and Evaluation (ADDIE)” stages is used in developmental research. Among the different educational design models, the ADDIE model stands out as highly renowned [23]. This model’s popularity stems from its comprehensive integration of epistemological, behaviorist, and constructivist theories, encompassing diverse viewpoints on learning. It is recognized as a universal model capable of accommodating various learning styles, whether traditional or digital. This basic development cycle was used in this study. Ethics committee approval for this study was obtained from Hacettepe University Ethics Committee (35,853,172/431.10–1677).

### Participants of the study

The study group consisted of experts in health sciences, software/testing specialists, educational technology specialists, assessment and evaluation specialists, and medical students. The study group was determined using a convenient sampling method. Student participation was voluntary and did not affect the grading, attendance, etc. in normal educational processes.

### Process

**Fig. 1:**
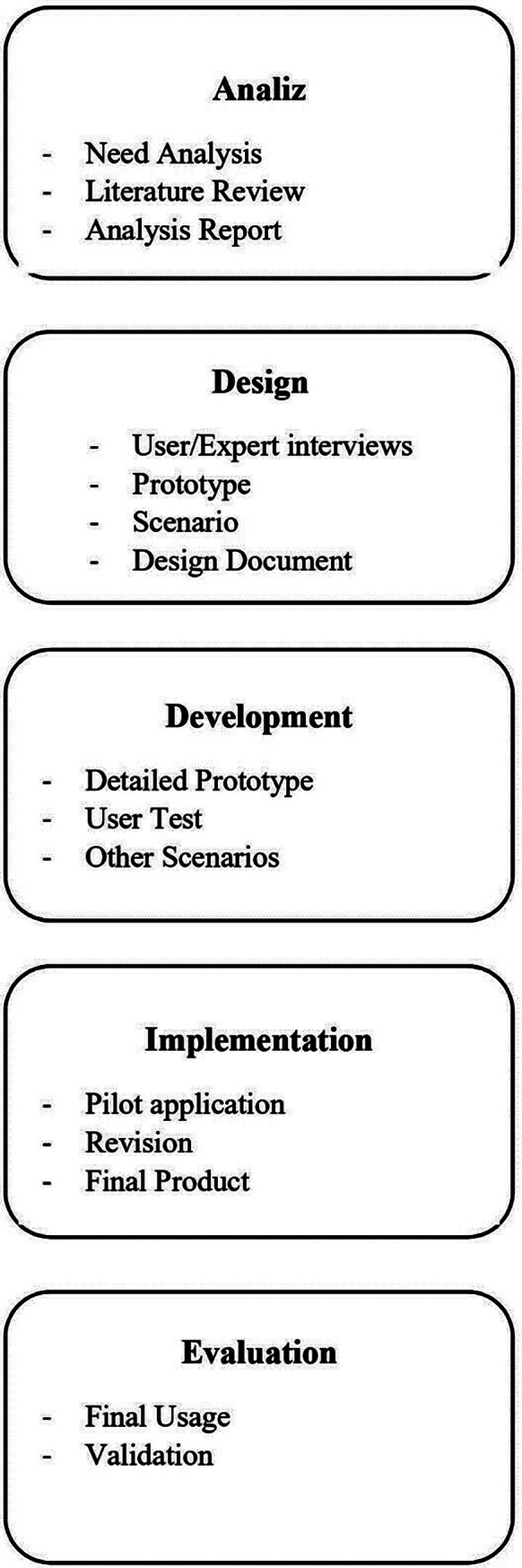
Process of the study

The following stages were carried out according to the product development cycle of the developmental research method. Figure 1 provides a concise overview of the process.

#### 1st stage (analysis)

At this stage, hard copies of ‘Needs Analysis’ form were distributed to the students (N = 50) and the lecturers working in the field of health (N = 4), to determine the requirements for the application. This form was used to collect information about the VP application that is to be designed in the next stage. VP application examples in the literature were examined and combined with the needs analysis form data and turned into an Analysis Report for the next stage.

#### 2nd stage (design)

At this stage, the analysis report of the previous stage was discussed and the Mockups of the VP application were created. Preliminary preparations were made for which subheadings (Introduction, Current Disease History, Past Disease History, Social History, Summary, and Closing) might be included in a patient interview and which questions should be asked under these subheadings were elaborated in a few sessions with the working group consisting of 50 medical students (hard copy scenario forms were distributed). Approximately 300 pages of question types were obtained by this activity. At the end of this stage, a Design Document was created about access to the application, interface, evaluation, reporting, etc.

#### 3rd stage (development)

At this stage, the prototype was prepared in line with the design stage data. First, the infrastructure, agent creation, assignment of movement /voice to the agent, and database creation (questions to be asked by users and the response of the VP) were carried out for the first -the “Headache”- scenario. Then system feedback was received for this first scenario (15 students participated). In line with these logs, script-matching studies were carried out, new concepts were created for the non-working questions, the questions leading to the same answer were connected to different concepts and the system interface was prepared according to the logs/opinions. Following these operations, the other 5 scenarios were also developed.

#### 4th and 5th stages (implementation and evaluation)

At this stage, after students used the VP application holistically, their usage logs and communication skills performances were evaluated through the system. This section is described in more detail under the heading of findings.

### The conversation engine and agent design

During the analysis phase of the study, first of all, the literature was searched and the findings of various studies related to the VP were examined [7, 8, 24–26]. The issues to be considered when creating and using VPs, which were highlighted in the literature, were discussed during the pertinent stages of the study, and the subsequent utilization of three core design elements took place.

- Visual reality (animation quality, 3D environment, mouth movement-sound harmony, image clarity, character creation relevant to culture, creating a virtual environment suitable for the previous experiences of the users) was carefully discussed in the Design and Development phase.
- Elements of behavioral reality (sound quality, adaptation of voice intonation to the responses given, VP establishing eye contact with the user, VP being a character that can move) were provided by various programs during the Development phase.
- Effective scenario (creating scenarios from good example practices currently used in training, presenting them to the opinions of experts at each stage, updating the scenario by conducting a pilot study, and preparing it according to a certain flow) was prepared.

On the other hand, the opinions and suggestions of 40 students were received via the needs analysis form. Then, interviews with experts were carried out and thematic analysis of open-ended items was performed and converted into frequencies. In this direction, the following decisions were made:

- Users were provided with short text information about the VP application during the first login to the system (f = 22),
- A contact page was prepared where students can express their opinions about the system (f = 19),
- Students were given instant feedback about their performance at the end of the application (f = 29),
- The system logs were ensured to be only accessible to the users determined by the admin (f = 27),
- When the system was asked a question that was not understood, the system continued with the most appropriate paired question in its tree structure (f = 24),
- The application allowed both written and verbal communication (f = 31),
- In the case of the questions that the system could not perceive, it was set to perceive the closest question and produced an answer (f = 8).

On the other hand, the VP application was decided to have 6 different scenarios (headache, kidney pain, ear pain, stomach pain, digestive problem and cold), and each scenario was decided to flow in 6 streams as ‘Introduction, Current Disease History, Past Disease History, Social History, Summary and Closing’, in line with the interviews conducted with the experts. After the analyses, a MockUp was created that allows interaction with screen-based written and verbal communication, can give feedback to the student during and after the practice, allows repetitive practices, and gives feedback on the user behaviors after practice as the application is compatible with multiple platforms. Then the next stage, the Design stage, was started.

At the beginning of the design stage, samples of questions to be asked to the patient in the scenario categories (Introduction, Social Story, Closing, etc.) for each scenario were collected from 50 students and menu designs were made. Since all possible question types were taken from the students to enrich the database and make the system more understandable, a Word document of 300 pages was integrated into the system for a total of 43 menus in 6 categories. This information in the database was then connected to a total of 3394 concepts as 21154 words (for example, in the introduction, the questions that can be asked as ‘How you’ve been?, Howdy-do?, Are you okay?, Can I find out how you are?, etc. instead of the question ‘How are you?‘, were all connected to the ‘How are you?‘ concept) Thus, the matches were completed to give more accurate answers to the questions. A usage scenario was then created (Table 1).

**Table 1:** Usage scenario

1. The user is presented with the application interface after logging in to the application. The following should be on this page: - Audio-Written communication area, - Agent field, - The algorithm that shows the stages of patient communication (Introduction-Current Disease History-Past Disease History-Social Story-Summary-Closing) and the progress of these stages (there are a certain number of questions that need to be asked at each stage, progress is shown in the relevant stage as these questions are asked) - A ‘help link’ in an appropriate area (what kind of questions should be asked, how long the interview should take, how it should end, etc.) - A ‘Note-Taking’ link in the appropriate area so that the student can take notes of the information he/she obtained during the interview. - End Interview (When this link is clicked, confirmation should be asked for exit and then directed to the Reporting page)
2. Reporting page - After the conversation is over, the user’s previous attempts/conversations can be presented as dates, success points, and duration. - Performance feedback for the user’s current conversation can be given numerically in percentages, which will be calculated by the trainer based on the total number of questions/correct number of questions asked. - An exit button on the reporting page can provide checking out from the application. - The administrator should be able to access logs such as how many users, which users, total time, score, etc. on the reporting page.

The menus in each scenario were scored by the experts according to the necessity and importance of asking the questions. This scoring was associated with the Communication Skills Evaluation Form and the total score that can be taken in each scenario was determined as 100. Students were enabled to collect points for each relevant correct question and this progress was instantly reflected on the screen graphically with visuals (scores were not reflected).

After the architecture of the application was established in the design phase, it was decided to develop the application in C# programming language. The services were created using ABP Suite™. The integration of the application with the natural dialogue tree and the operation of the matching algorithm with the menus in the tree was established. Scripts were created to ensure that the case selected on the case page searches the tree by asking questions to the relevant tree and returns announcements by providing matches. The CrazyTalk™ program was used for the agent’s mouth movements. All situations in the design phase were coded and the system prototype was created.

Besides, a field to take notes was created that allows practicing students to take instant notes during the interview. An application service was written in which reports (logs) could be presented and the relevant data layers were prepared. Activities for case solutions were presented as a reporting page. In the report, it was ensured that the columns include the case practiced, the user who solved the case, the number of correct questions, the score received, the transaction date, and the type of case termination (for the cases that were left in half / not completed). Filtering was conducted according to the parameters in the list. The actual implementation and evaluation phase were started after these procedures.

### Data collection tools

Data were collected according to different requirements at each stage of the research. The data collection tools and process of the research are presented below.

#### Needs analysis form

This form, which is created by the authors and finalized with the experts. Information about what a possible VP application should cover, how the interface should be, how to access the interface, sub-headings of the scenarios, expectations/suggestions, etc. was obtained by this form. The form consisted of eight questions; three open-ended questions were evaluated by conceptual content analysis, and the answers to the other five multiple-choice questions were evaluated by percentage/frequency.

#### Scenario building form

Firstly, the subheadings of scenarios determined by the experts (Introduction, Current Disease History, Past Disease History, Social History, Summary, and Closing) that were to be included in the VP interviews were presented to the students in a blank document. An example of a possible question in each subheading was shared with the students and the students were asked to create question variants (e.g. under the sub-heading of ‘Current Disease History’, suggestions of students on the different versions of asking the question of ‘What is your complaint?‘ can be requested). Thus, a total of 56 questions for a scenario and a 300-page document were created. The VP database is strengthened with possible question types in this way.

#### System validation form

This form, which is created by the authors and finalized with the experts, consists of 13 questions, and aims for the verification of whether the developed application is the valid and the desired product or not, by the students. Questions are about whether the application is a user-friendly and effective product, from its agent to its guidelines. These questions and the evaluations of the students are presented in the findings section.

#### Communication skills assessment form

This form is currently being used to evaluate the communication skills training with SPs in the faculty where the research is conducted. The maximum score that can be obtained from this form, which consists of a total of 12 questions (with Yes-No options), is 100.

#### Interview form

The authors created this form to get the opinions of the students who use the final version of the application. In this form, three open-ended questions were asked about the strengths, weaknesses and aspects of the application that needed strengthening.

### Analysis of data

The data obtained in the study were analyzed by using qualitative and quantitative data analysis techniques. Descriptive statistics were used in the analysis of qualitative (conceptual analysis was used) and quantitative data.

## Results

The prototyped system was used as a pilot application by the students of the Faculty of Medicine (N = 15) and by the experts for the user validation of the first scenario (headache). The most important update after these applications was ‘concept improvement’. This improvement ensured that when a question was asked, the logs in cases that lead to more than one answer were evaluated and these questions were directed to the same concept so that they could result in the ‘right answer’. The accuracy values for the prototype (1 scenario) and the original product after revision (6 scenarios) are presented in Table 2.

**Table 2:** VP dialogue accuracy

	Prototype	Prototype	Final Application	Final Application
Dialogue type	Typed	Spoken	Typed	Spoken
Students	n = 4	n = 11	n = 56	n = 142
Total questions asked	165	412	1641	4058
Answered correctly, %	55.3	60.4	81.1	85.2
Answered incorrectly, %	12.2	14.3	5.6	4.8
Not answered, %	32.5	25.3	13.3	10

As can be seen in Tables 2 and 4 out of 15 students communicated in written form and 11 verbally with the VP version (prototype- headache scenario) before the final product was developed. Students asked 165 questions in writing and 412 questions orally. These questions were answered correctly in the ratios of 55.3% (typed) to 60.4% (spoken), answered incorrectly (pattern mismatched) in the ratios of 12.2% (typed) to 14.3% (spoken), and could not be answered in the ratios of 32.5% (typed) to 25.3% (spoken) (no pattern matched). The other 5 scenarios were developed by the concept improvement studies, and each student used a different scenario. After the revision, the percentages of success increased to 81.1% (typed) and 85.2% (spoken) (Table 1). Concordantly, the percentage of questions that could not be answered or answered incorrectly also decreased due to the increase in this ratio.

On the other hand, the implementation phase involved the one group post-test quasi-experimental design. Both during the prototype development process (n = 15) and during the creation of the final application (n = 198), the communication skills scores of the students were evaluated through the logs. The increase in the correct answer percentages of the system naturally caused the students to ask the right questions and the performance score of communication skills increased. This mean score was 36.74 (maximum score was 100) in the interviews in which 15 students participated before the revision. The mean score in the interviews carried out in the final product was determined as 74.2 (maximum score 100, p < 0.001). Following this application, the system was opened to the students (n = 26) after 3 weeks to allow a group of students to practice repeatedly, and they made a new interview with the VP on the relevant scenario they had previously made. At the end of this practice, students were found to conduct 17% more effective interviews compared to their first interview in terms of communication skills mean score (mean score: 89.2, p < 0.001).

During the evaluation of the final product, the data obtained from the students’ ‘Validation Form’ (n = 198) were evaluated and it was observed that students generally thought the VP application was developed in accordance with the purpose, it was user-friendly, but although the scenario was sufficient, they expected a higher level of application supported by artificial intelligence (AI). Information on the data collected is presented in Table 3.

**Table 3:** Student feedback

Items	Mean	SD
1. The application generally serves its purpose.	4.56	0.72
2. The application supports students to communicate effectively with the applicant/patient in the future.	3.61	0.96
3. The application interface is user-friendly.	4.77	0.52
4. The application’s ability to give feedback to users is sufficient.	3.92	0.65
5. The interview scenario in the application is appropriate.	4.66	0.55
6. The virtual patient agent in the application is realistic.	3.69	0.61
7. When communicating with the virtual patient by voice, the application’s speech detection level is at the appropriate level.	4.06	0.65
8. When communicating with the virtual patient in writing, the level of perception of what is written by the application is at the appropriate level.	4.15	0.48
9. The application is supportive in developing communication skills.	3.61	0.74
10. I have no difficulty in using the application.	4.88	0.26
11. The instructions in the application are sufficient.	4.22	0.43
12. The help menu in the application is sufficient to meet the needs that may arise before/during use.	4.44	0.50
13. I recommend this application to others.	4.69	0.42

The weaknesses, strengths and areas of potential improvement of the system, and the thematic codes created in the light of these findings, which were determined by the feedback received from the students who used the last product (n = 85) via the Interview Form, are presented in Table 4. The authors generated the codes by analyzing the process evaluations provided by the students at the conclusion of the course.

**Table 4:** Thematic codes for virtual patient application

Weaknesses			Strength			Areas of Potential Improvement		
Code	f	%	Code	f	%	Code	f	%
Not being a real patient	30	60	Repetitive Practice	44	37.9	Mutual Communication	16	42
Internet Problem	12	24	Visual Realism	30	25.9	Artificial Intelligence	16	42
Inability to recognize typos	8	16	Powerful Scenario	22	19	Speed	6	16
			Instant Feedback	20	17.2			

As can be seen in Table 4, students generally stated the strengths of the application as follows: creating the chance of ‘Repetitive Practice’, which is the most important feature of the simulation; moving-3D visual and the harmony between lip movements and sound; the comprehensiveness of the scenario and giving instant feedback to the students visually after each question they ask. However, students thought that it would be more effective to make dual conversation (the VP should also ask questions to the physician candidate) and stated that the system not automatically correcting mistakes resulting from delays in the connection of the users and typos from time to time, as the weaknesses of the application. It was also seen that the students expected a fast system that operates with AI, perceives the question regarding the type of questions, and responds accordingly.

## Discussion

In this study, a VP was developed to improve the ability of medical students to take histories within the scope of communication skills training. The VP developed in this study was developed in line with the data obtained by literature search, expert opinion, and needs analysis. Considerations when creating and using VPs, that are highlighted in the literature [24–26] were discussed in the relevant stages of this research. In this context, ‘Visual Reality’, ‘Behavioral Reality’, and ‘Effective Scenario’ aspects were evaluated by experts and students at all stages of the study and an optimum product was created. In general, reflecting the aspects which are highlighted in the literature and specified by the experts and students during the analysis phase, such as the students being able to communicate both verbally and in writing, taking instant notes during the interview, receiving instant feedback on performance, rapid reaction of the system, and submission of a performance report at the end of the application, is thought to cause the VP application to be effective in communication skills training.

The VP developed within the scope of the study was first tested with a prototype and the system was allowed to be more powerful with the revisions made in line with the obtained logs. Processes such as concept matching, updating the questions leading to repeated answers in the database, and linking the unanswered questions to new concepts during these revisions improved the application. Thus, script matching increased from 58% in the first application to 83% in the final application. This helped students to ask the right question at the right time. For example, in the first-time application with 15 students, the mean score of communication skills assessment in the interviews was 36.74 (out of 100 points), while in the final application with 198 student interviews, the mean score was 74.2. After 3 weeks, the students who used the system again (n = 26) made 17% more effective interviews compared to their first practice (score: 89.2). This situation indicates that the application was successfully improved with revisions and the repeated practice, which is one of the most important features of the VP application; hence contributed to a performance increase. These findings are also consistent with literature studies suggesting that VP use improves the communication skills of students in medicine [7, 9, 27], pharmacy [25], nursing [8], and dental [28] fields of health sciences.

The categorization of scenarios based on a flow and the system’s capacity to provide immediate feedback for each student input were found to enhance the application’s effectiveness. Although the physician candidates who participated in the research had experienced VPs for the first time, they gave positive feedback such that this application was motivating, especially because of the innovation potential, that it let them remember the basic history-taking questions because it gave instant feedback, and that it allowed patient-physician interviews to be held at any time.

In Turkey, SPs are generally used in teaching communication skills. However, this situation has some disadvantages as stated in the literature [7, 8, 17, 29]. These are reported as Consistency-Forgetting Lines, Adaptability, Manpower, Cost, Versatility-Elicits Bias, and Role Model [7, 9]. VHs provide unique benefits that real humans do not provide. Users’ experiences can be better standardized with VHs than with human beings [30]. VPs can be used 24/7 and can be presented to students at any role/character (pediatric, obese, physical deformities, etc.) according to the scenario in a very short time. Students have the experience of gaining a limited number of communication skills with SPs, in the country where this research was conducted. However, this experience can be easily provided continuously at any time, with VP applications.

## Conclusion

In conclusion, the VP application developed in this study was shown to improve the history-taking skills of medical students within the scope of communication skills. VPs, which are characterized by availability at any time/place/number, easy accessibility, and the ability to communicate both in writing and verbally, can be a good complement to existing communication skills training. Such VPs can help students in health sciences to be well-prepared for real clinical settings. It is recommended to develop and use AI-supported VPs that can establish both communication and examination skills together.

### Limitations

Our integrated artificial intelligence system would be even more captivating. This would allow the most appropriate response to be pulled up among the responses stored in the database. The VP application could potentially enhance its effectiveness by engaging with students through two-way interactions and posing questions to them. Greater effectiveness can be attained by creating offline systems that mitigate the impact of sudden internet disruptions.

### Advice to practitioners

- These applications can be integrated into various faculties by creating Virtual Human study groups, under the cooperation of health science-social sciences.
- Various scenarios specific to health sciences (nursing, physical therapy, pharmacy, etc.) can be created and cost-effective solutions can be introduced.
- Since it is difficult to find SPs who speak English at the national level, the script and voice-over of the VP application can be translated into English to support many international students to communicate effectively.

### Recommendations for researchers

- It can be investigated whether VPs that communicate verbally or those that communicate in writing have a different effect on the success and satisfaction of students.
- AI technologies can be employed, wherein the VP can also pose questions, or the system can automatically rectify instances where students omit questions, and more.
- How VP practices create a behavioral change in clinical environments in the real lives of students can be investigated by carrying out long-term follow-up studies.

## Data Availability

The datasets used and analyzed during the current study are available from the corresponding author on request.
